# An Eight-Gene Blood Expression Profile Predicts the Response to Infliximab in Rheumatoid Arthritis

**DOI:** 10.1371/journal.pone.0007556

**Published:** 2009-10-22

**Authors:** Antonio Julià, Alba Erra, Carles Palacio, Carlos Tomas, Xavier Sans, Pere Barceló, Sara Marsal

**Affiliations:** 1 Grup de Recerca de Reumatologia, Institut de Recerca Hospital Universitari Vall d'Hebron, Barcelona, Spain; 2 Unitat d'Hematologia, Hospital Universitari Vall d'Hebron, Barcelona, Spain; Deutsches Krebsforschungszentrum, Germany

## Abstract

**Background:**

TNF alpha blockade agents like infliximab are actually the treatment of choice for those rheumatoid arthritis (RA) patients who fail standard therapy. However, a considerable percentage of anti-TNF alpha treated patients do not show a significant clinical response. Given that new therapies for treatment of RA have been recently approved, there is a pressing need to find a system that reliably predicts treatment response. We hypothesized that the analysis of whole blood gene expression profiles of RA patients could be used to build a robust predictor to infliximab therapy.

**Methods and Findings:**

We performed microarray gene expression analysis on whole blood RNA samples from RA patients starting infliximab therapy (n = 44). The clinical response to infliximab was determined at week 14 using the EULAR criteria. Blood cell populations were determined using flow cytometry at baseline, week 2 and week 14 of treatment. Using complete cross-validation and repeated random sampling we identified a robust 8-gene predictor model (96.6% Leave One Out prediction accuracy, *P* = 0.0001). Applying this model to an independent validation set of RA patients, we estimated an 85.7% prediction accuracy (75–100%, 95% CI). In parallel, we also observed a significantly higher number of CD4+CD25+ cells (i.e. regulatory T cells) in the responder group compared to the non responder group at baseline (*P* = 0.0009).

**Conclusions:**

The present 8-gene model obtained from whole blood expression efficiently predicts response to infliximab in RA patients. The application of the present system in the clinical setting could assist the clinician in the selection of the optimal treatment strategy in RA.

## Introduction

Rheumatoid Arthritis (RA) is one of the most prevalent autoimmune diseases in the world and is characterized by the chronic inflammation of the synovial joints. In RA, the sustained inflammatory process progressively destroys the articular cartilage and subchondral bone leading, in many cases, to major functional disability [1]. The origin of the disease is unknown but it is actually accepted that it is caused by the complex interaction of a genetic susceptibility background and environmental factors [2], [3]. This lack of knowledge, however, has not prevented the development of pharmacological treatments that can efficiently control the progression of the disease. The clearest exponent of this success has been the treatment of active RA through the neutralization of Tumor Necrosis Factor alpha (TNF-alpha) cytokine.

Infliximab was the first TNF alpha blocker for RA treatment to be clinically tested and is a genetically constructed IgG1 murine-human chimeric monoclonal antibody that binds both to the soluble subunit as well as the membrane-bound precursor of TNF-alpha [4]. Like the other two approved anti-TNF blockers, adalimumab and etanercept, infliximab has proven to be an efficacious treatment for disease activity control in patients with RA [1]. There is however a subgroup of RA patients (ranging 20–40%) who, in spite of synovial TNF-alpha production do not respond to general TNF blockade therapy [5]. In these cases, alternative approved biological therapies like rituximab or abatacept, would be more beneficial for the patient [6]. Therefore, a major goal in RA treatment is to identify a reliable response predictor to anti-TNF alpha therapy.

Several systems have been assayed for the prediction of clinical response to anti-TNF alpha in RA patients. The most direct approach, the measurement of clinical variables at the beginning of treatment, has been found to be a weak predictor of the type of response [7]. For this reason, the search for informative biomarkers is actually been favoured. None of the biomarkers analyzed to date has been found to have sufficient predictive power to be useful in the practical setting [8]. However, the recent introduction of high-throughput analytical systems is now expected to be a major breakthrough in biomarker discovery. In particular, gene expression microarrays have already shown to be a very powerful technology for the identification of gene expression profiles predictive of disease evolution or treatment response [9], [10].

In the present study we have used microarray technology for the identification and validation of a whole blood gene expression profile predictive of the response to infliximab in RA patients. With the implementation of a standardized processing methodology and the use of robust analysis methods, we have identified a powerful 8-gene model predictor for the response to infliximab treatment in RA patients.

## Methods

### Patients

From January 2005 to June 2007, those RA patients with active disease (i.e. defined as a Disease Activity Score (DAS28) >3.2) from the Rheumatology Unit of the Hospital Universitari Vall d'Hebron (Barcelona, Spain) that were going to start infliximab therapy were considered for inclusion in the present study. The DAS28 score is a combined index to measure the disease activity in patients with RA and it has been extensively used in clinical trials as well as daily clinical practice [11]. It uses the number of tender and swollen joints (from a total of 28 joints), a measure of systemic inflammatory activity (erythrocyte sedimentation rate in this case), as well as a general health assessment of the patient using a visual analog scale. The inclusion criteria for RA patients in the present study were 1) fulfilment of the revised 1987 American Rheumatism Association criteria [12] 2) naïve to anti-TNF alpha treatment 3) receiving concomitant metothrexate (MTX) treatment of ≤20 mg/wk or maximum tolerable and 4) concomitant therapy with prednisolone (GC, dose ≤10 mg/day or equivalent) and NSAID 5) having stable MTX, GC and NSAID doses during the previous 4 weeks to the inclusion in the study 6) having discontinued previous DMARDs at least 4 weeks prior to the inclusion 7) fulfilment of all standard inclusion criteria defined for infliximab treatment. Exclusion criteria were 1) patients positive for hepatitis B or C virus (active or inactive) 2) fulfilment of all standard exclusion criteria defined for infliximab treatment. All patients signed an informed consent at the time of enrolment, and all the procedures followed were in accordance with the ethical standards of the Institut de Recerca Hospital Universitari Vall d'Hebron ethics committee and with the Helsinki Declaration.

### Clinical procedures

Clinical variables associated with disease activity were recorded at the time of the first infliximab infusion (wk0) and at weeks 2 and 14 of treatment. The assessment of the clinical response to infliximab therapy was performed at week 14 using the European League Against Rheumatism (EULAR) criteria [13]. The EULAR criteria is the reference method for determining the response to treatment in RA that combines both an absolute value of disease activity (DAS28) as well as the relative improvement [14]. With this method patients are classified into good, moderate or poor response. This method uses two variables 1) the baseline DAS28 score (i.e. week 0 in this case) and 2) the variation in the DAS28 activity score due to treatment (i.e. variation between week 14 and week 0). In the present study we used a binary outcome variable: “poor” response individuals were classified as “non-responders”, and “moderate” or “good” response individuals were classified as “responders”.

### Blood collection and RNA extraction

Blood samples were extracted at weeks 0 (RNA and flow cytometry analyses), 2 and 14 (flow cytometry analyses only) of infliximab treatment from all patients. All blood RNA samples were obtained, preserved and extracted following a well-defined protocol we specifically developed for this study (Supporting Information S1). Blood RNA was preserved at the same time of venipuncture using the PAXgene Blood RNA System (PreAnalytix, Switzerland). Samples were store frozen at −80°C until RNA isolation. Total RNA was extracted using the PAXgene RNA Isolation kit (Qiagen, Valencia, CA, USA) following the recommended protocol. We extracted the RNA only from those patients that reached the time of treatment response determination (i.e. week 14, n = 44 patients). The RNA extraction was performed simultaneously in all samples to minimize the technical variation associated with this step and RNA integrity was assessed using the Nano total RNA kit from the 2100 BioAnalyzer system (Agilent, Santa Clara, CA, USA).

### Microarray analysis

Whole-genome gene expression analysis was performed using the Illumina Human-6 v1 Beadchip array system (Illumina, San Diego, CA, USA). This microarray platform measures the gene expression levels of more than 47,000 transcripts using 50-mer DNA probes fixed on a bead-based system. At the same time the microarray data was being acquired, an updated version of the Illumina Beadchip was launched. This new version included a redesign of several probe sequences, principally, those not belonging to the RefSeq database. Therefore, we restricted the microarray analysis only on those probes that were still considered as valid (Illumina in-house data, available online).

Only good quality blood RNAs (i.e. 28S/18S ratio close to 2, RNA Integrity Number >8) were subsequently processed using the Illumina gene expression assay. Biotin-labeled cRNA was hybridized to Sentrix whole genome beadchips and scanned on the Illumina BeadStation 500x. The raw intensity data was obtained from the scanned arrays using the BeadStudio software version 1.4.02 (Illumina, San Diego, CA, USA), using the default probe summarization and background substraction methods. The statistical analyses were performed using the open-source statistical environment “R” and the associated Bioconductor project libraries for genomic analyses [15].

The log2-transformed intensity values were normalized using the quantile normalization method [16] implemented in the *affy* package. Quality control analysis of the normalized data identified an outlying gene expression profile which was excluded from further analyses (Supporting Information S1). Before analyzing the normalized data, we performed a filtering step in order to exclude uninformative genes. Those probes for which all gene-expression values were under the lowest 5^th^ percentile of the global gene-expression values were considered as non-expressed and discarded (n = 4,150). Probes with a low variability (coefficient of variation <0.03, n = 14,701) were also removed. All microarray data is in accordance with MIAME guidelines and is accessible through GEO database reference GSE12051.

### Unsupervised analysis of RA gene expression patterns

Before building the response predictor, we sought to determine if the whole blood gene expression profiles at week 0 already clustered RA patients according to their response to anti-TNF alpha. Unsupervised classification techniques like hierarchical clustering analysis are suitable for this purpose. However, they are generally used without any assessment of the statistical robustness of the identified clusters [17]. In the present study we use the resampling-based method implemented in the Bioconductor package “clusterStab” to determine both the optimal number of clusters and the statistical significance of the final clustering [18]. The differential gene expression between the significant clusters was performed using the Welch's t-test implemented in the *multtest* package.

### Response predictor building and validation

**Figure 1:**
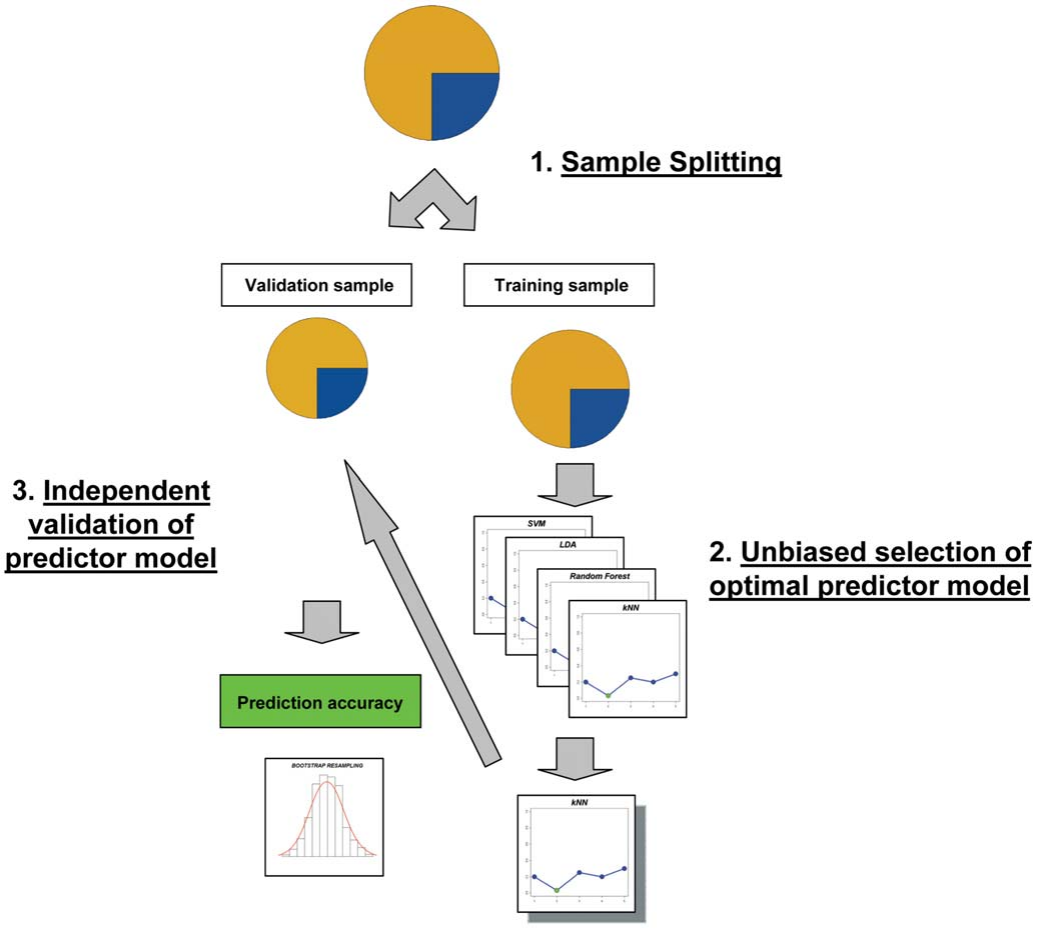
Methodology for building and validating a robust microarray predictor. The construction of robust microarray-based predictors must necessarily follow a series of steps in order to avoid analytical biases and ensure a real applicability of the model. First, the original sample is split in two subsamples: the training sample and the validation sample. In the training sample we seek to find the optimal classifier; complete cross-validation (leave-one out cross validation in our case) gives an unbiased measure of the power of each tested model. In the case that we find similarly good performing models, a resampling method (i.e. permutation testing) can be used to objectively select the most robust between them. Only once we have chosen the optimal model we will apply it to the validation sample. Since we have not used the information from this independent sample in building the predictor, the accuracy determined from this sample set is an optimal estimation of the power of the model in a real setting. A resampling method (i.e. bootstrap analysis) can be used to estimate the confidence intervals associated with the predictor accuracy.

Predictor building and validation (Figure 1) was performed using the established robust methodology for microarray prediction: first, the global sample was randomly divided into a training sample (2/3 of the sample, 29 patients) and a validation sample (1/3 of the sample, 14 patients). Given the moderate sample size, we used balanced sampling to ensure a similar proportion of responders and non-responders in both sample sets. In the training sample, we selected the optimal classifier method and its parameters through Leave-One-Out Cross Validation (LOOCV). This repeated cross validation method is a powerful means to evaluate the performance of a classifier without incurring in gene selection bias [19]. In particular, the predictor genes are independently determined at each round of cross validation without using the left-out sample. The new model is then applied to this external sample to obtain an independent estimate of the predictor's accuracy.

In the present study we evaluated Support Vector Machines, Diagonal Discriminant Analysis (Diagonal Linear Discriminant Analysis or DLDA and Diagonal Quadratic Discriminant Analysis or DQDA), Random Forests and *k–*Nearest Neighbours using the Bioconductor libraries *e1071*, *ipred*, *randomForest* and *class*, respectively. Details on the parameters used in the classifier evaluation are included in Supporting Information S1.

In order to select the final model between equally good predictor models we calculated a permutation *P* value associated with their prediction accuracy. This step avoids model selection bias, that is, to select simpler models only based in their lower complexity [20]. Finally, we used the independent validation set to determine the accuracy of the predictor. Bootstrap resampling was used to calculate the 95% confidence intervals associated to the estimated precision accuracy using the percentile estimation method [21].

### Flow cytometry analysis

In all RA patients included in the study, we performed cell cytometry analyses the same day of blood extraction for microarray analysis. We determined the main leukocyte subpopulations (i.e. neutrophils, lymphocytes and monocytes) as well as red blood cell (RBC) and platelet counts. Within the lymphocyte subpopulation, we performed a complete evaluation of different subsets including CD3+8+, CD3+4+, CD4+CD8+ (i.e. double positive lymphocytes), CD4+CD28+ (i.e. active CD4+ T cells [22]) and CD4+CD25+ (i.e. regulatory CD4+ T cells [23]). Briefly, cells were stained by direct immunofluorescence using monoclonal antibodies conjugated with fluorochromes FITC, Phycoerythrin, Pycoerythrin-cyanin 5 and ECD. Isotype-matched immunoglobulins with no reactivity against surface markers and the fluorochrome combination were used as negative controls. After antibody incubation and subsequent erythrocyte lysis, we performed cell count acquisition using the EPICS-XL MCL (Coulter, Germany). Statistical significance was assessed using t-test or paired t-test when appropriate. In the study of cell cytometry changes along time, those individuals with missing data in any of the three time points (i.e. week 0, 2 and 14) were excluded from the paired analysis.

## Results

### Assessment of clinical response of infliximab treated patients

During the 2 year-recruitment period 48 RA patients with active disease starting infliximab therapy were selected for the present study. From these, 44 patients reached week 14 of treatment whilst 4 were withdrawn (for septic arthritis, myocardial infarction, infusion reaction and voluntary discontinuation in each case). Using the binary classification described previously 7 patients were categorized as “Non-Responders” and 37 as “Responders” (Table 1).

**Table 1:** Characteristics of RA Patients.

	Global	Responders	Non-Responders	*P*-value*
**Age (years)**	52.73±11.37	51.84±10.85	57.43±13.77	0.34
**Sex (women:men)**	38 5	32:4	6 1	0.57
**RA duration (years)**	14.32±9.82	13.04±9.04	19.0±12	0.30
**DAS28 week 0**	5.94±1.17	5.981±1.20	5.76±1.05	0.62
**DAS28 week 14**	4.09±1.18	3.83±1.07	5.44±1.047	0.0002
**CCP (% positive)**	86.05%	86.11%	83.33%	0.99
**RF (% positive)**	58.14%	55.55%	71.42%	0.68

Plus-minus values are means ± SD. The DAS28 score was defined according to the European League against Rheumatism criteria. *P-value calculated using Welch's t-test or Fisher's exact test when appropriate.

### Unsupervised analysis of baseline gene expression profiles

The optimal clustering number parameter was found to be k = 2 (Supporting Information S1). Whilst one of the patient clusters was statistically stable (86% of 1,000 resamplings the original cluster appears) the other grouping of patients appeared to be relatively unstable (40% of 1,000 resamplings the original cluster appears). We also found that unsupervised classification at baseline did not segregate patients according to their treatment response (Supporting Information S1).

In order to understand the origin of the observed patient grouping we performed the differential gene expression analysis between both clusters. Although Gene Ontology (GO) analysis did not show any statistically overrepresented GO term (data not shown), we could identify several erythrocyte-related genes overexpressed in one of the clusters (Supporting Information S1). We subsequently compared the blood cell counts and haemoglobin concentration between both groups and found that the latter was statistically significant (mean haemoglobin concentration of cluster 1 = 13.05 g/l vs. mean of cluster 2 = 11.41 g/l, *P* value  = 0.0008, Supporting Information S1). Importantly, this confounder was not present when comparing haemoglobin concentrations between patients classified according to their response to infliximab (*P* = 0.71).

### Microarray predictor building and testing

**Figure 2:**
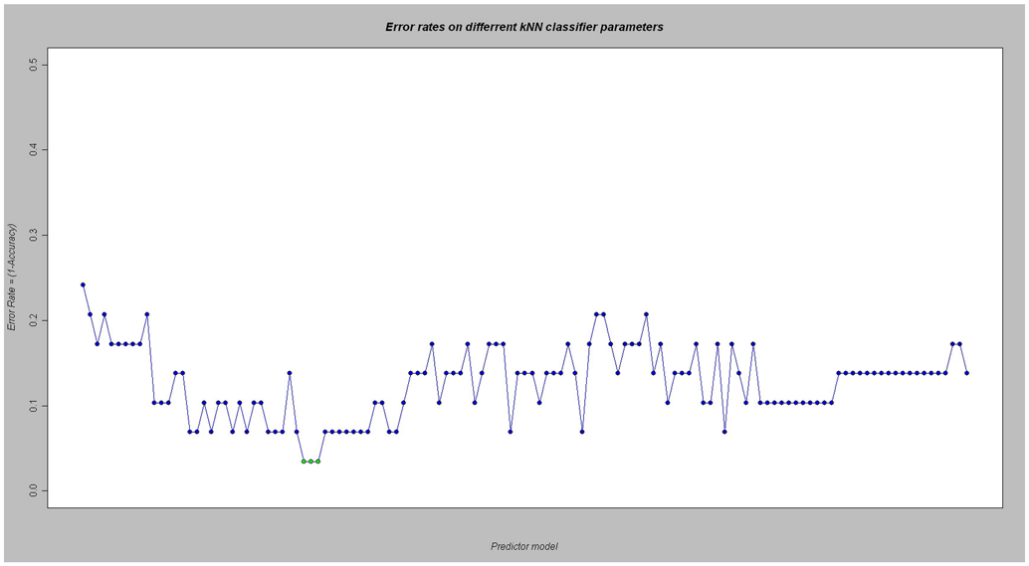
Error rates associated to different parameter values for kNN classifier. From left to right, predictor models with increasing number of genes and increasing number of nearest-neighbours are evaluated in the training dataset using LOOCV. The 8 gene model under 3, 4 or 5 nearest neighbours (green color) were found to be the optimal classifiers with only 1 patient misclassified out of 29 (0.034 error rate).

After applying the gene filtering steps, a total of n = 3,364 genes were selected for the microarray predictor building and testing. The exhaustive analysis of different classifier algorithms using the Leave One Out Cross-Validation method showed an overall good predictive performance (Supporting Information S1). Using either Support Vector Machines, Linear Discriminant Analysis, Random Forests or k-Nearest Neighbours (kNN) classifiers, the best prediction accuracy attainable was, at least, of 89.6%. From these, the kNN classifier reached a nearly perfect prediction accuracy (1 misclassification out of 29, 96.6% prediction accuracy) in an 8 gene model using either 3, 4 or 5 nearest neighbours (Figure 2). The only misclassified individual in the LOOCV analysis was a responder individual that was incorrectly classified as a non-responder. All infliximab non-responder patients were correctly predicted.

In order to select the final classifier between the equally performing kNN models, we calculated a permutation *P* value for the prediction accuracy. All three models were highly significant, confirming the robustness of the classifier method (*P* = 0.0001, *P* = 0.0002, and *P* = 0.0003, for 3-, 4- and 5- nearest neighbours models, respectively. We finally selected for validation the 3-nearest neighbour kNN model, since it had the most significant *P* value.

**Figure 3:**
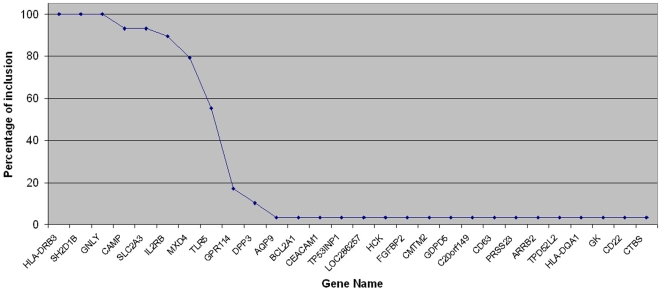
Percentage of inclusion of all genes selected through LOOCV. The present plot shows the percentage that each gene is selected amongst the top 8 genes after 29 rounds of LOOCV. It can be seen that, from all genes, the 8-predictor gene group is systematically selected indicating a strong correlation with the outcome. The remaining genes seem to be selected on a random basis.

We next sought to identify the consistency of each of the predictor genes throughout the different cross validation steps (Figure 3). We found that 6 of the 8 predictor genes -HLA-DRB3, SH2D1B, GNLY, CAMP, SLC2A3 and IL2RB- were selected more than 80% of the time, and the remaining 2 -MXD4 and TLR5- were selected more than 50% of the time. This finding clearly demonstrates the strong correlation between the gene expression of each predictor gene with the response outcome.

### Validation of the microarray predictor

Applying the final 8 gene predictor model in and independent dataset of 14 patients, 12 of them were correctly classified indicating an 86% prediction accuracy. Bootstrap determination of the 95% confidence interval for the estimated prediction accuracy was 75 to 100%. The incorrectly classified individuals were 1 infliximab responder and 1 non-responder, thus giving an estimated sensitivity and specificity of 91.6% and 50%, respectively. Taking into account the performance of the predictor both in the LOOCV and in the external validation, we estimated a 94.4% sensitivity and an 85.7% specificity of the present predictor model.

### Flow cytometry analysis of blood cells according to infliximab response

**Figure 4:**
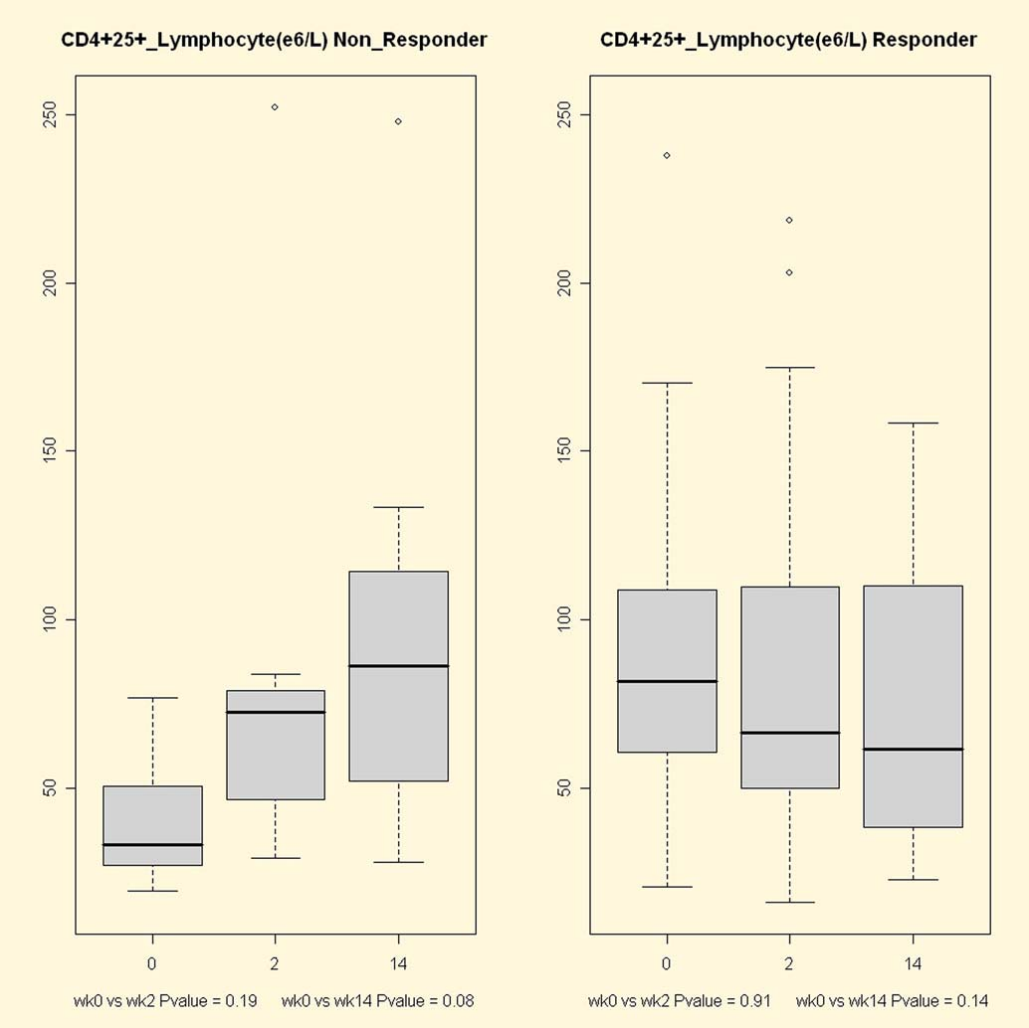
Flow cytometry CD4+CD25+ lymphocyte counts from responders and non responders to infliximab at weeks 0, 2 and 14. Non responders had a significantly lower CD4+CD25+ lymphocyte fraction than responders at baseline (*P* = 0.0009). During the treatment this CD4+ subpopulation increased, ending with similar levels to the responder group.

Comparing the flow cytometry measures between response groups at each treatment time, we found responders to have a significantly higher number of CD4+CD25+ T lymphocytes at baseline (*P* = 0.0009, Figure 4). We also found that, in this group, the number of neutrophils at week 2 was significantly lower compared to the non responder group (4.1 e9/L vs. 2.9 e9/L, respectively, *P* = 0.032; see Supporting Information S2).

The analysis of the flow cytometry changes associated to anti-TNF alpha treatment identified a major effect in the responder group. In this group, platelet and neutrophil counts showed a highly significant reduction along the two treatment weeks (*P*<0.001 in all cases). Response to infliximab was also associated with a reduction in peripheral monocytes but only at week 2 of treatment (*P* = 0.03). No statistically significant variation was observed in the CD4+ lymphocyte compartment, although an increasing trend in CD4+CD25+ cell numbers could be observed in the non-responder group (Figure 4).

We evaluated the use of CD4+CD25+ as predictors of anti-TNF alpha therapy using generalized linear model fitting. However, the performance of the identified predictor was not as good as the microarray gene expression predictor (71% prediction accuracy, 50% specificity and 75% sensitivity).

## Discussion

Using whole blood gene expression profiles of RA patients we have built and validated a robust predictor of response to infliximab therapy. We have identified an eight gene expression model with a leave-one out cross validation accuracy of 96.5% and an independent validation accuracy of 86.5% (CI: 75–100%). In parallel, we have found a significantly higher number of baseline CD4+CD25+ T cells in the responder group compared to the non-responder group (*P*<0.0009).

The process of building a robust gene expression predictor has two main steps: 1) unbiased selection of the predictor model parameters and 2) estimation of the predictor model generalisability [19]. In the present study we have taken into account these two fundamental steps in the building and validation of the microarray predictor to infliximab therapy. First, using complete cross-validation we have identified the optimal model using the kNN algorithm. The high prediction accuracy already determined at this step is an unbiased measure of the strong predictive power of the model. Another important measure of the prognostic value of a microarray predictor is the stability of the predictive genes [24]. We have found that the group of 8 predictor genes is preferentially selected at each independent round of cross validation demonstrating the strong correlation between gene expression and the type of response; some genes –HLA-DRB3, SH2D1B and GNLY-, are even selected at all rounds. Second, we have confirmed the generalisability of the predictor by applying it to an independent validation set. The confidence interval for the prediction accuracy was relatively narrow, emphasizing the good power of the model to predict the response to anti-TNF alpha therapy. Taking together the estimates in the internal (LOOCV) and external validation, the present predictor has an estimated 94.4% sensitivity and 85.7% specificity for the prediction of infliximab response which is the highest performance reached by a biomarker for this therapy to date.

To date, levels of systemic TNF alpha, Rheumatoid Factor, anti-CCP antibodies and C-Reactive Protein have been analyzed for their association with treatment response, but the results have shown either inconsistent or with no practical use in the clinical setting [8]. Also, several genetic markers like TNF alpha promoter SNPs or the shared epitope have been tested for their predictability but with yet inconclusive results [25], [26]. In the present study we have used the whole blood gene expression profile as a biomarker for infliximab response prediction. Blood is an ideal biomarker tissue both for being noninvasive and for its practical application in the clinical settings [27]. Although a relatively complex tissue, whole blood gene expression has been shown to be informative of particular disease conditions [28], [29]. In RA, previous studies using blood mononuclear cell isolates and microarray technology have been performed in the search for a response predictor to anti-TNF therapy [30], [31], [32]. However, none of these have entirely followed the robust predictor methodology described here and therefore their findings should be considered with caution [19]. In the present study we show that, whilst unsuitable for unsupervised classification, the whole blood gene expression can be useful for the efficient prediction of a response to a particular treatment, in this case, systemic anti-TNF alpha blockade. In the present study we have also developed and implemented a standardized methodology which should facilitate its extended use in different clinical settings.

The study of patients segregated according to anti-TNF alpha response can be useful to identify relevant biological mechanisms in RA [5]. We have found that infliximab responders have a higher number of CD4+CD25+ T cells (i.e. Tregs) than non-responders at baseline. Tregs are powerful regulators of autoimmune responses and there is increasing evidence for their implication in several autoimmune disorders including RA [33], [34]. Recently, a new mechanism involving this cell type has been proposed for anti-TNF therapy effect [34]. In this model, TNF blockade would elicit the formation of *de novo* Tregs from peripheral CD4+CD25- effector lymphocytes, which would in turn reduce the proinflammatory activity of the disease. However, we have not found a significant increase in this CD4+ compartment in the response group along treatment; this tendency is rather observed in the non-responder group. From our observations, it seems that response to infliximab is more favourable under higher numbers of this cell type at baseline. Interestingly, one of the predictor genes overexpressed in non-responders, IL2RB, has been also found to be significantly overexpressed in peripheral mononuclear cells of infliximab non-responders at baseline [32]. IL2RB, together with CD25 (also known as IL2RA), forms a medium affinity receptor for the lymphocyte growth factor IL2. Specific studies are needed to define the precise origin of this differential gene expression and its relation to treatment response. The remaining predictor genes have all been linked to the immune response or even to autoimmunity [35], [36], [37], [38], [39] but, to date, this is the first evidence of their association to anti-TNF alpha treatment response.

The present study is an important step towards the management of RA through molecular biomarkers. Recently, new therapies for the control of RA have been developed and several others are soon going to become available. With this increase in treatment options, there is a pressing need to find systems that reliably predict the efficacy of a particular treatment for a particular patient. The present 8-gene predictor model for infliximab treatment can be a powerful tool for therapy individualization in RA and future studies should be carried out to transpose these results into usual health-care settings.

## Supporting Information

Supporting Information S1 (0.30 MB DOC)

Supporting Information S2 (0.17 MB DOC)
