# Supplementary material for: An Eight-Gene Blood Expression Profile Predicts the Response to Infliximab in Rheumatoid Arthritis
Source: PLoS One. 2009 Oct 22;4(10):e7556. doi: 10.1371/journal.pone.0007556 (PMC2762038; doi:10.1371/journal.pone.0007556)
Supplement: Supporting Information S1 — (0.30 MB DOC) [file pone.0007556.s001.doc]

Supporting information Text S1: Protocol for PaxGene Blood RNA conservation and extraction

In the present study, we followed the protocol for standardization of sample collection and conservation:

**Sample standards**

- Blood was collected from RA patients the same day of the first infusion of infliximab therapy and before the anti-TNF infusion.
- According to Whitney *et al*. the time of collection of the blood sample if one of the most influencing factors in gene expression profiles.^1^ All blood samples were specifically collected at the same time frame (between 8:30-9:30 a.m.).
- All patients need to be fasting at the time at blood collection.
- Blood samples for cytometry analysis were taken at the same time as the sample for RNA extraction.

**Blood collection and preservation**

- Blood was extracted using the PaxGene tube (PreAnalytix, Switzerland; reference number: #762165). This system preserves RNA profiles since the same moment of patient venipuncture. Immediately after extraction, the tube was inverted 20 times for the appropriate sample mixing with the preserving agent.
- The PaxGene tube was kept at room temperature until 13:00 a.m. and then kept at 4ºC for 6-8 hours.
- Finally, the PaxGene tube was vertically frozen at -80ºC and kept until RNA extraction.

**Total RNA extraction and quality control analysis**

- Before RNA extraction, PaxGene tubes were left to unfreeze at room temperature overnight (~ 14 hours).
- Total RNA was extracted following the PaxGene Blood RNA Kit protocol (Qiagen, USA; reference number: 762174)
- RNA concentration was measured using Nanodrop UV spectrometer (Nanodrop Technologies, Delaware, USA) and RNA integrity was evaluated using the RNA 6000 Nano Assay (Agilent, California, USA; reference number: 5065-4476). Only good quality RNAs (i.e. 28S/18S ratio close to 2, RNA Integrity Number > 8) were subsequently analyzed for gene expression profiling.
- RNA was kept at -80ºC until processing with Illumina Beadchip following the Illumina gene expression assay (Illumina, San Diego, CA, USA).

1. Whitney AR, Diehn M, Popper SJ, et al. Individuality and variation in gene expression patterns in human blood*. Proc Natl Acad Sci U S* A 200**3;1**00(**4**):1896-901.

**Figure S1. Identification and removal of gene expression outlier in normalized data.** After performing quantile normalization of all 44 microarrays, using hierarchical clustering as quality control tool we identified one microarray with an outlying expression profile (average Pearson correlation coefficient < 80%). The MA plot analysis of this individual confirmed the outlying gene expression pattern (data not shown). Given the lack of outlying clinical or blood cytometry data for this individual (data not shown), this variation is most probably due to a gross technical artefact rather than true biological variation.

**Figure S2**. **Optimal number of clusters as estimated with clusterStab method.** It can be seen that the cluster number that obtains a higher number of bootstrap resamplings with Jaccard coefficients close to 1 is k=2 (n=1,000 bootstrap resamplings).

**Figure S3: Unsupervised classification on whole blood RNA profiles of RA patients at week 0 of infliximab treatment.** Clustering analysis on baseline gene expression using the optimal clustering number did not aggregate patients according to their future response to infliximab treatment. The left cluster (cluster 1) was found to have a good stability (86% of n=1,000 bootstrap resamplings) but the right cluster (cluster 2) was found to be less reproducible (40% of n=1,000 bootstrap resamplings). R: Responder, NR: Non Responder.

**Table S1. List of top differentially expressed genes between the two clusters of RA patients obtained after unsupervised analysis of their blood gene expression profiles. The most significant genes (*P-*value < 1E-7)** are sorted according to their statistical significance. GID: Illumina probe ID; Pvalue: Bonferroni-corrected significance value for differential expression between clusters; FCh: Fold Change (Cluster 2 – Cluster 1).

| **GID** | **Pvalue** | **FCh** | **Gene_Name** | **Accession** | **Definition** |
| --- | --- | --- | --- | --- | --- |
| GI_21614516-A | 1.00E-12 | -1.16 | **GYPC** | NM_016815.2 | H. sapiens glycophorin C (Gerbich blood group) (GYPC), transcript variant 2, mRNA. |
| GI_17921996-S | 8.96E-12 | -1.08 | **MYL4** | NM_002476.2 | H. sapiens myosin, light polypeptide 4, alkali; atrial, embryonic (MYL4), mRNA. |
| GI_4503580-S | 3.66E-11 | -1.51 | **EPB49** | NM_001978.1 | H. sapiens erythrocyte membrane protein band 4.9 (dematin) (EPB49), mRNA. |
| GI_21361518-S | 4.33E-11 | -1.13 | **ADIPOR1** | NM_015999.2 | H. sapiens adiponectin receptor 1 (ADIPOR1), mRNA. |
| GI_34147566-S | 8.89E-11 | -0.93 | **GUK1** | NM_000858.3 | H. sapiens guanylate kinase 1 (GUK1), mRNA. |
| GI_21359970-S | 1.10E-10 | -1.22 | **ALS2CR2** | NM_018571.4 | H. sapiens ALS 2 (juvenile) chromosome region, candidate 2 (ALS2CR2), mRNA. |
| GI_20336333-A | 4.97E-10 | -1.09 | **BCL2L1** | NM_001191.2 | H. sapiens BCL2-like 1 (BCL2L1), transcript variant 2, mRNA. |
| GI_18201877-S | 7.11E-10 | -1.33 | **C20orf108** | NM_080821.1 | H. sapiens chromosome 20 open reading frame 108 (C20orf108), mRNA. |
| GI_31542848-S | 8.22E-10 | -1.46 | **GMPR** | NM_006877.2 | H. sapiens guanosine monophosphate reductase (GMPR), mRNA. |
| GI_7705631-S | 2.06E-09 | -0.70 | **TTC11** | NM_016068.1 | H. sapiens tetratricopeptide repeat domain 11 (TTC11), mRNA. |
| GI_37539300-S |  5.37E-09 | -1.11 | **LOC378204** | XM_353706.1 | H. sapiens ILP-interacting protein ILPIPA (LOC378204), mRNA. |
| GI_22218618-S | 8.45E-09 | -1.06 | **KIAA1892** | NM_015397.1 | H. sapiens KIAA1892 (KIAA1892), mRNA. |
| GI_18860914-S | 1.40E-08 | -0.77 | **XK** | NM_021083.2 | H. sapiens Kell blood group precursor (McLeod phenotype) (XK), mRNA. |
| GI_6806896-I | 1.48E-08 | -1.03 | **SNCA** | NM_000345.2 | H. sapiens synuclein, alpha, transcript variant NACP140, mRNA. |
| GI_31377590-S | 2.40E-08 | -0.99 | **SESN3** | NM_144665.2 | H. sapiens sestrin 3 (SESN3), mRNA. |
| GI_7706179-S | 2.52E-08 | -1.62 | **ERAF** | NM_016633.1 | H. sapiens erythroid associated factor (ERAF), mRNA. |
| GI_34222220-S | 3.54E-08 | -0.92 | **IMP-2** | NM_006548.3 | H. sapiens IGF-II mRNA-binding protein 2 (IMP-2), mRNA. |
| GI_16306549-S | 4.08E-08 | -1.64 | **SELENBP1** | NM_003944.2 | H. sapiens selenium binding protein 1 (SELENBP1), mRNA. |
| GI_42516575-S | 5.37E-08 | -1.15 | **C14orf87** | NM_016417.2 | H. sapiens chromosome 14 open reading frame 87 (C14orf87), mRNA. |
| GI_36030882-S | 6.88E-08 | -0.49 | **RP42** | NM_020640.2 | H. sapiens RP42 homolog (RP42), mRNA. |
| GI_8923856-S | 6.99E-08 | -0.53 | **LOC55831** | NM_018447.1 | H. sapiens 30 kDa protein (LOC55831), mRNA. |
| GI_34452680-S | 8.90E-08 | -0.80 | **RNF10** | NM_014868.3 | H. sapiens ring finger protein 10 (RNF10), mRNA. |


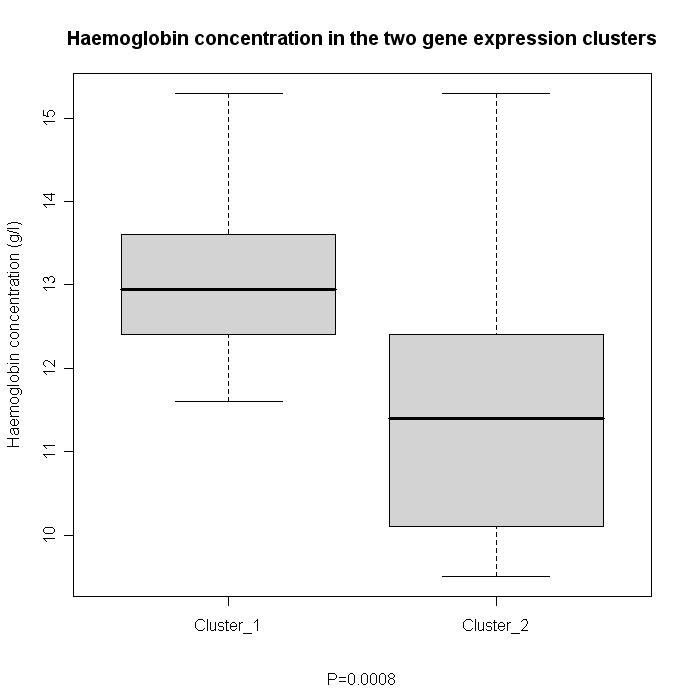
 (**A)**


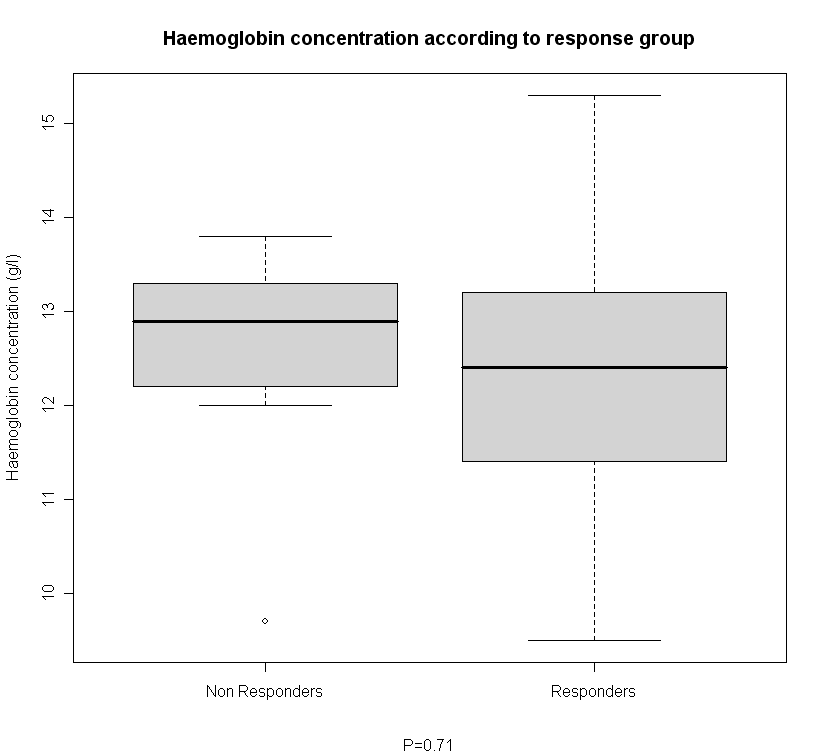
 **(B)**

**Figure S4. Haemoglobin concentration differences in RA patients grouped according to unsupervised analysis clustering and according to treatment response.** It can be clearly seen that hierarchical clustering of blood RNA profiles of RA patients is highly correlated with the differences in the levels of haemoglobin concentration (*P* = 0.0008) (A). This confounding effect is not observed when comparing haemoglobin levels between responders and non-responders (*P* = 0.71) (B).

Supporting information Text S2: Analysis of different classifier algorithms for prediction to infliximab response

Using LOOCV we evaluated different machine learning classifiers under different parameter combinations. One common parameter for microarray predictor building is the number of genes used: we evaluated different numbers of the F-test best ranked genes (2 to 20, 40, 50, 70, 100 and 150 top genes). The evaluated classifier methods and parameters were:

- Support Vector Machines (SVMs, *e1071* package): we evaluated both the Radial Basis Function and Polynomial kernels.
- Diagonal Discriminant Analysis (*ipred* package): covariance matrices can be assumed to be constant across the classes (i.e Linear Discriminant Analysis, DLDA) or they may vary across the classes (i.e. Quadratic Discriminant Analysis).
- Random Forests (*randomForest*  package): the number of trees was set to 5000 andwe used the default number of variables randomly sampled as candidates.
- K-Nearest Neighbours (*class* package): 2 to 10 nearest-neighbours were evaluated,

The next series of plots represent the LOO cross-validation error rate (1-prediction accuracy) of several of the models tested. In each graphic, the number of genes used in the predictor increases from left to right and, for each n number of genes, specific classifier parameters are evaluated. In green are highlighted those predictor models with an error rate < 0.1. The red line depicts the best error rate value (0.03) which is only obtained using the kNN classifier algorithm (8 gene model with 3 to 5 nearest neighbours).
