# Supplementary material for: An Eight-Gene Blood Expression Profile Predicts the Response to Infliximab in Rheumatoid Arthritis
Source: PLoS One. 2009 Oct 22;4(10):e7556. doi: 10.1371/journal.pone.0007556 (PMC2762038; doi:10.1371/journal.pone.0007556)
Supplement: Supporting Information S2 — (0.17 MB DOC) [file pone.0007556.s002.doc]

**Index of citometry figures**

**Boxplots: Non Responders vs. Responders at each treatment week (t-test P-value)**

- Red blood cell (e12/L) ………………………………………………………………………………………………………. Page 2
- Platelets (e9/L) ………………………………………………………………………………………………………………. Page 3
- Leukocytes (e9/L) ……………………………………………………………………………………………………............ Page 4
- Neutrophils (e9/L) …………………………………………………………………………………………………………… Page 5
- Lymphocytes (e9/L) …………………………………………………………………………………………………………. Page 6
- Monocytes (e9/L) …………………………………………………………………………………………………………… Page 7
- CD3+ Lymphocytes (e9/L) …………………………………………………………………………………………………. Page 8
- CD4+ Lymphocytes (e9/L) …………………………………………………………………………………………………… Page 9
- CD8+ Lymphocytes (e9/L) …………………………………………………………………………………………………… Page 10
- CD4+8+ Lymphocytes (e6/L) ………………………………………………………………………………………………… Page 11
- CD4+28+ Lymphocytes (e6/L) ………………………………………………………………………………………………. Page 12
- CD4+25+ Lymphocytes (e6/L) ………………………………………………………………………………………………. Page 13

**Boxplots of temporal evolution for Responders and for Non Responders (paired t-test P-value: wk 0 vs. wk2 and wk 0 vs. wk14)**

- Red blood cell (e12/L) ………………………………………………………………………………………………………. Page 14
- Platelets (e9/L) ………………………………………………………………………………………………………………. Page 15
- Leukocytes (e9/L) ……………………………………………………………………………………………………............ Page 16
- Neutrophils (e9/L) …………………………………………………………………………………………………………… Page 17
- Lymphocytes (e9/L) …………………………………………………………………………………………………………. Page 18
- Monocytes (e9/L) ……………………………………………………………………………………………………………. Page 19
- CD3+ Lymphocytes (e9/L) ………………………………………………………………………………………………….. Page 20
- CD4+ Lymphocytes (e9/L) …………………………………………………………………………………………………… Page 21
- CD8+ Lymphocytes (e9/L) …………………………………………………………………………………………………… Page 22
- CD4+8+ Lymphocytes (e6/L) ………………………………………………………………………………………………… Page 23
- CD4+28+ Lymphocytes (e6/L) ………………………………………………………………………………………………. Page 24
- CD4+25+ Lymphocytes (e6/L) ………………………………………………………………………………………………. Page 25
